# Perineal Abscess Following SpaceOAR Insertion

**DOI:** 10.7759/cureus.51050

**Published:** 2023-12-24

**Authors:** Umair Azhar, Justin Lin, Rahman Sayed, Zaki Masoud, Aroosa Zamarud, Ravinder Kaler

**Affiliations:** 1 Department of Radiology, Albert Einstein College of Medicine, New York, USA; 2 Internal Medicine, Northwell Health, New York, USA; 3 Department of Neurosurgery, Albert Einstein College of Medicine, New York, USA; 4 Internal Medicine, Albert Einstein College of Medicine, New York, USA; 5 Department of Neurosurgery, Stanford Health Care, Palo Alto, USA

**Keywords:** drainage, prostate cancer, radiation, abscess, spaceoar hydrogel

## Abstract

This case report discusses a 64-year-old male who presented with a perineal abscess following the insertion of the SpaceOAR hydrogel, highlighting a rare but potentially serious complication of the hydrogel. Hydrogel spacers have become integral in prostate cancer radiotherapy by reducing rectal toxicity. Ensuring proper technique, prophylactic antibiotics, and vigilant post-insertion monitoring are crucial for averting complications. This case underscores the significance of early diagnosis and management in preventing severe consequences and emphasizes the need for a high index of clinical suspicion when patients present with post-insertion symptoms.

## Introduction

Rectal hydrogel spacers are used in radiotherapy for prostate cancer and allow the administration of higher radiation doses to the prostate while minimizing radiation-induced rectal toxicity. SpaceOAR hydrogel (Boston Scientific Corporation, Marlborough, MA, USA) made up of water and polyethylene glycol is designed as a perirectal spacer; the hydrogel is placed between the prostate and the rectum via a syringe inserted through the perineum and the placement is confirmed using ultrasound imaging [1]. The use of hydrogel spacers has been evaluated in several studies, and their safety and efficacy have been established [2-4]. However, we have only found one documented detailed case report on abscess complications following SpaceOAR hydrogel insertion [5]. In this report, we describe a patient who presented with a perineal abscess following SpaceOAR insertion.

## Case presentation

A 64-year-old man presented to our hospital with perineal pain for approximately two weeks in duration. He had a history of prostate cancer for which he recently discontinued 50 mg of bicalutamide daily and is scheduled to begin leuprolide acetate (Lupron) injections. His other significant medical history included hypertension and hyperlipidemia, well controlled by atorvastatin 10 mg daily and aspirin 81 mg daily. The patient reported a recent SpaceOAR hydrogel placement for prostate radiation therapy.

On examination, the patient was febrile at 38.7^o^C along with a heart rate of 95 and a BP of 136/64 mmHg. His abdomen was soft, nontender, and nondistended with normoactive bowel sounds. He had a nontender prostate and a mildly swollen scrotum. A Foley was placed in the emergency department (ED) for acute urinary retention.

Investigations

**Figure 1:**
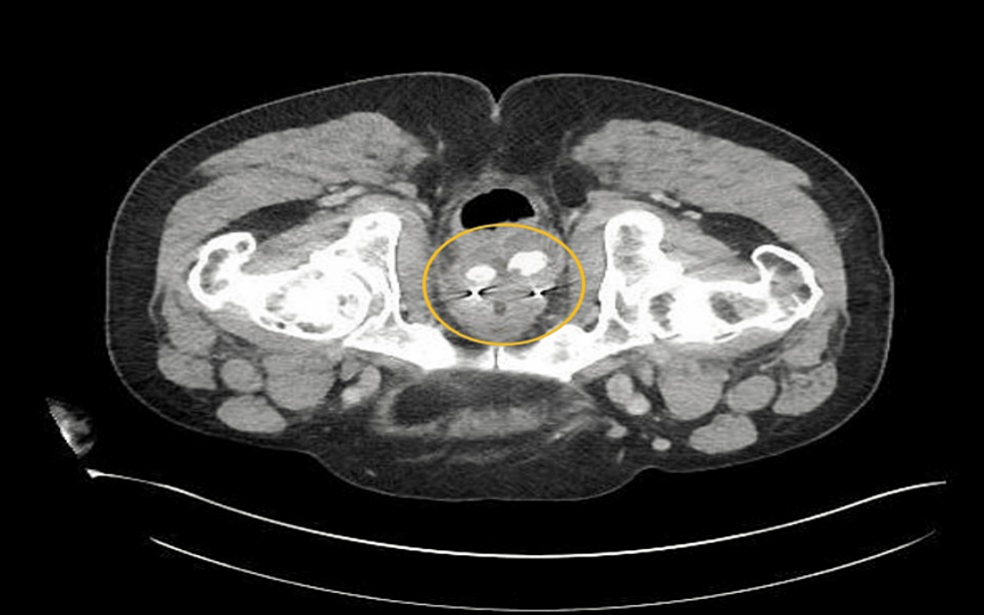
CT of the pelvis with contrast (axial view) Rim-enhancing collection (shown within the yellow circle) measuring 5.7 x 3.5 x 5.4 cm with internal areas of high attenuation between the prostate and rectum correlating with the location of SpaceOAR hydrogel placement with increased extension inferiorly into the perineum.

**Figure 2:**
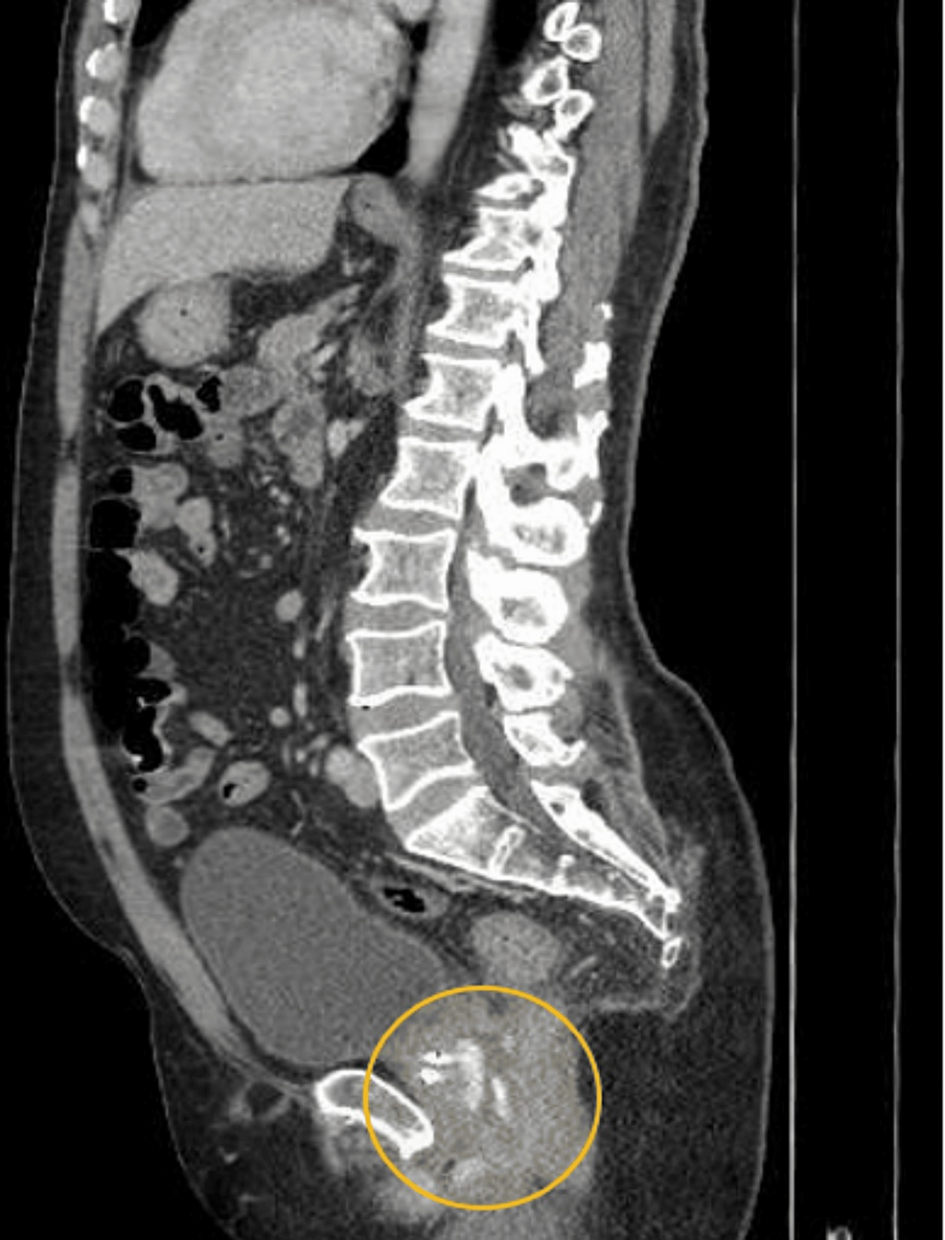
CT of the pelvis with contrast (sagittal view) Moderate soft tissue inflammation at the perineum with a 3.2 x 5.3 x 5.0 cm heterogeneous complex fluid collection (shown within the yellow circle) located between the prostate and the rectum. There are areas of hyper-density within the collection as well as a small hyper-dense focus within the perineal soft tissues. The location of the collection is consistent with hydrogel placement.

Investigation revealed the patient to be anemic, with hemoglobin and hematocrit of 11.5 g/dL and 33.9%, respectively. The patient also had leukocytosis, with white blood cell levels of 16.49 x 103 uL. Axial CT scan of the abdomen and pelvis showed moderate soft tissue inflammation at the perineum with a 5.7 x 3.5 x 5.4 cm heterogeneous complex fluid collection located between the prostate and the rectum (Figure 1). The same fluid collection can be seen again on sagittal view (Figure 2). There were also areas of hyper-density within the collection as well as a small hyper-dense focus within the perineal soft tissues. The location of the collection was consistent with hydrogel placement.

Management

The patient was started on intravenous vancomycin and piperacillin-tazobactam for empiric antibiotic coverage. He was then taken by the urology team for surgery. Incision and drainage of the abscess were performed via the perineum with 150 cc of purulent fluid drained, irrigation was performed with betadine and hydrogen peroxide, the wound was packed, and a Penrose drain was left in the perineum. General surgery was consulted intraoperatively to evaluate the rectum and found no fistula or perforation. No further debridement was performed. Post-operatively, the patient noticed an immediate and substantial improvement in pain. Abscess gram stain initially revealed rare gram variable coccobacilli; however, abscess and blood cultures ultimately had no growth, likely in the setting of concurrent antibiotic coverage. The patient was transitioned over to oral amoxicillin/clavulanic acid for a total of seven days of antibiotics post-operative infection control and discharged home. He was subsequently followed by the urology department for drain removal and had a complete resolution of symptoms.

## Discussion

The insertion of a hydrogel rectal spacer between the prostate and rectum is an innovative procedure allowing for higher doses of radiation in the treatment of prostate cancer. By creating a space between the prostate and rectum, the risk of improper radiation to the rectum is mitigated, which continues to be regarded as dose-limiting toxicity [4]. While long-term studies have not been completed, as of now, the short-term effects of rectal spacers have shown to be safe with minimal complications [2]. In 2021, Vaggers et al. completed a systematic review of glycol-based hydrogel rectal spacers. They found that the rate of infection was 6% with two doses of 500 mg oral ciprofloxacin. After adjusted antibiotic prophylaxis with ceftriaxone and gentamicin, no further infections were observed [6]. One study reported rectal infiltration with associated recto-urethral fistula further resulting in osteomyelitis; however, this study demonstrates the importance of proper technique with proper follow-up imaging as opposed to a safety issue with the hydrogel spacer itself [7]. Other studies reported self-limiting rectal discomfort and pain, which resolved within one week of insertion [8].

In our case report, we describe a patient who presented with a perineal abscess with heterogeneous complex fluid collection and areas of hyper-density only weeks after SpaceOAR hydrogel placement and starting radiation therapy. The patient’s clinical picture of active perineal discomfort and fever suggested an infectious etiology. Prostatic abscesses should be treated promptly with antibiotic treatment and may be combined with drainage if needed. While not always necessary, surgical drainage has been shown to limit the duration of antibiotics needed, shorten hospitalization, and enhance voiding function [9]. Conservative management using only antibiotics is usually acceptable for abscesses less than 1 cm in size whereas larger abscesses >2 cm in diameter had a better response with surgical intervention.

## Conclusions

The use of hydrogel spacers in radiotherapy for prostate cancer has become a novel technique in reducing rectal toxicity in recent years. Proper techniques and prophylactic antibiotic treatment should be strictly implemented to prevent complications. A high index of clinical suspicion must be maintained for anyone presenting with symptoms such as fever, pain, or discharge after hydrogel spacer placement. These symptoms may indicate rare but serious complications such as abscess formation due to hydrogel spacer infection. Investigation with CT is required for prompt diagnosis and subsequent management, which can help prevent devastating complications for the patient.
